# Direct-to-Consumer Genetic Tests and Canadian Genetic Counselors: A Pilot Exploration of Professional Roles in Response to Novel Biotechnologies

**DOI:** 10.3390/genes15020156

**Published:** 2024-01-25

**Authors:** Cassandra E. Haley, Ma’n H. Zawati

**Affiliations:** Centre of Genomics and Policy, McGill University, Montreal, QC H3A 0G1, Canada; cassandra.haley@mail.mcgill.ca

**Keywords:** direct-to-consumer genetic testing, DTC-GT, genetic counseling, health literacy, healthcare resource utilization, ethics

## Abstract

The role of genetic counselors is evolving in response to health-related direct-to-consumer genetic tests (DTC-GT). While there is consensus in the literature that pre- and post-DTC-GT genetic counseling would benefit consumers, genetic counselors have reservations about DTC-GTs, and there is a paucity of research on providing DTC-GT counseling. This pilot quantitative survey is the first study to examine Canadian genetic counselors’ views on DTC-GTs and how this disruptive biotechnology affects their role, and consumer informed consent and privacy. Canadian genetic counselors are cognizant of the harm to informed consent and privacy associated with DTC-GT, but are hesitant to engage directly, wary of misusing clinical time and resources. However, counselors are open to producing educational materials on DTC-GTs and collaborating with other stakeholders and the DTC-GT industry to support consumers. In this study, practical considerations for DTC-GT counseling sessions are discussed, including the unique needs of DTC-GT patients and the challenges posed by DTC-GTs to the genetic counseling duty to inform. This research benefits genetic counselors and physicians by examining how best to utilize genetic counselors’ skills in the DTC-GT context, to minimize burdens on the healthcare system and support DTC-GT consumers.

## 1. Introduction

Direct-to-consumer genetic testing (DTC-GT) for health purposes grants consumers unprecedented access to their health data by directly providing information on one’s disease risks or genetic susceptibilities, with the implicit assumption that such knowledge could lead to changed lifestyles and health behaviours among consumers [1]. However, the removal of healthcare providers, and particularly genetic counselors (GCs) from the discussions of genetic and genomic health pose great concern for consumer informed consent and the interpretation of results. Studies have noted that DTC-GT companies lack pre-counseling, fail to include a comprehensive overview of the risks and benefits, present challenges to healthcare data privacy, and lack transparent policies regarding the return of incidental findings [2,3,4,5], all of which present a serious concern for ensuring informed consent at purchase. A recent confidentiality breach in a leading DTC-GT company further serves to underscore the magnitude of the privacy and confidentiality concerns relating to DTC-GTs [6].

DTC-GTs are contentious within the literature for a variety of reasons, not limited to their public perception as a diagnosis and propensity toward the misunderstanding of the results by the public [7,8,9] who may lack an understanding of the risks [10], and the tendency of the results to be emotionally distressing [11,12,13] and confusing as algorithms update the results [14]. DTC-GTs may also be misconceived as medical grade, clinically valid, and scientifically accurate, which could push consumers to pursue clinical validation with subsequent strain on healthcare systems and providers, or may lead to the delay of important diagnoses, owing to a false sense of security from a test [3,7,9,10,11,12,15,16,17,18,19,20].

There is consensus in the literature that GCs ought to be involved in the pre- and post-counseling of DTC-GTs to mediate the public’s direct access to these technologies, to encourage informed consent at purchase, and to guide consumers through understanding the results of the tests [3,8,9,18,21,22,23,24,25,26,27].

However, there is a paucity of recent research regarding how the services of GCs ought to be best utilized in the context of DTC-GTs. A 2018 survey of Canadian and American GCs reported that a majority (89.7%) of respondents agreed or strongly agreed that DTC-GT companies should provide in-house counseling, while 41% of respondents reported feeling uncomfortable providing counseling to DTC-GT consumers due to a lack of knowledge needed to counsel on the results, uncertainty around the accuracy of DTC-GTs, and such counseling being a misuse of clinical time [23].

In light of the general skepticism of Canadian GCs towards DTC-GTs [26], an online quantitative survey was developed to explore the concerns of practicing GCs, their opinions on the various policy directives within the corporate DTC-GT community, and what they envision their role to be in the industry. With the findings of the survey, we suggest points to consider for Canadian GCs who are interested in engaging with DTC-GT genetic tests and patients who pursue DTC-GTs.

## 2. Materials and Methods

### 2.1. Instrumentation and Data Analysis

As this project involved interactions with healthcare professionals, study approval was obtained from the McGill University Faculty of Medicine Institutional Review Board in December 2021 (A12-B97-21B). Annual renewal was obtained in December 2022 and December 2023.

The online survey consisted of 48 broad closed-ended questions designed to take under 30 min to complete and was available in English and French to maximize the number of respondents. The survey objectives were to (1) describe the current role of GCs with regard to DTC-GTs, (2) compare alternate strategies of involvement of GCs with DTC-GTs, and (3) elucidate the potential implications of GCs engaging with DTC-GTs.

All the survey questions were analyzed through descriptive statistics.

### 2.2. Participants

Genetic counselors who held Canadian or American certifications and who were currently working with patients in Canada were included in this survey. A total of 26 responses were included in the survey, while only 25 completed responses were included in the data analysis. According to the Canadian Association of Genetic Counsellors (CAGC), the survey went out to approximately 400 members. The survey was active between 1 March 2022 and 15 April 2022.

### 2.3. Survey Limitations

Ultimately, this survey data, while useful for describing the preliminary sentiments of Canadian GCs regarding DTC -GTs, was not statistically significant. The CAGC advertises over 400 members [28], and only 25 members responded to this survey, representing a 6.25% response rate.

While this response rate was not statistically significant, the results of this survey still added value to the literature. This survey represents the first effort to understand Canadian GCs’ opinions on DTC-GTs, specifically interrogating their views on how DTC-GTs challenge important paradigms of the GCing vocation, including informed consent and privacy. To our knowledge, this survey is also the first to examine how this disruptive biotechnology impacts the traditional duties and roles of the Canadian GC, providing initial exploratory evidence for how Canadian GCs may view their changing roles as they adapt to unprecedented public interaction with genetic information.

The low response rate reported here could have been due to a number of factors, the most predominant being the ongoing COVID-19 pandemic. GCs have been overworked and expected to cope with higher-than-ever demands as the prevalence of genetics in healthcare has grown and waitlists have increased. It is, therefore, very plausible that GCs are prioritizing patient care over responding to surveys. The timing of the first recruitment email also coincided with the beginning of Spring break, and given that the field of GCing is 95% female [29], often with young families, the initial recruitment email may have been missed. A possible solution to this low participation rate could have been to extend the survey through several more months, but such an endeavour was beyond the timeline of the project.

Additionally, it is worth considering that electronic surveys are known to have a low response rate, and physicians (and by correlation GCs as healthcare workers) have lower response rates than the general public [30]. Indeed, the Canadian Medical Association’s 2018 National Physician Health Survey had an 8.5% response rate, which the authors noted was a typical response rate for online surveys [31]. Furthermore, while the 2020 National Society of Genetic Counselors (NSGC) Professional Status Survey was used to estimate the response rate, GCs may selectively answer this survey at a much higher rate than others, since the results of the survey directly impact their work and reflect the status of the entire profession.

Institutions that GCs work with may also have policies that artificially limited the response rate. Internal policies may be in place preventing GCs from accepting referrals for patients who engage with DTC-GTs. Therefore, GCs at these institutions may never encounter DTC-GT consumers and so decided not to respond to the survey.

It is also important to note that survey attrition is a common occurrence with quantitative surveys. The themes and topics explored in this survey were broad and respondents may have had more nuanced opinions than the format allowed. Therefore, the respondents may have had difficulty with the finite closed-ended structures and may have been unable to engage with the topic in such a way.

## 3. Results and Discussion

This pilot survey represents the first effort to understand Canadian genetic counselors’ opinions on DTC-GTs, specifically by interrogating their views on how DTC-GTs challenge important paradigms of the genetic counseling vocation, including informed consent and privacy. It is also the first to examine how this disruptive biotechnology impacts the traditional duties and roles of the Canadian genetic counselor as they adapt to unprecedented public interaction with genetic information. Additionally, this study aids genetic counselors and physicians in better understanding the role of genetic counselors and how to best utilize their professional skills around DTC-GTs in order to minimize burdens on the healthcare system and further the understanding of the support required by DTC-GT consumers.

### 3.1. Survey Results

**Figure 1:**
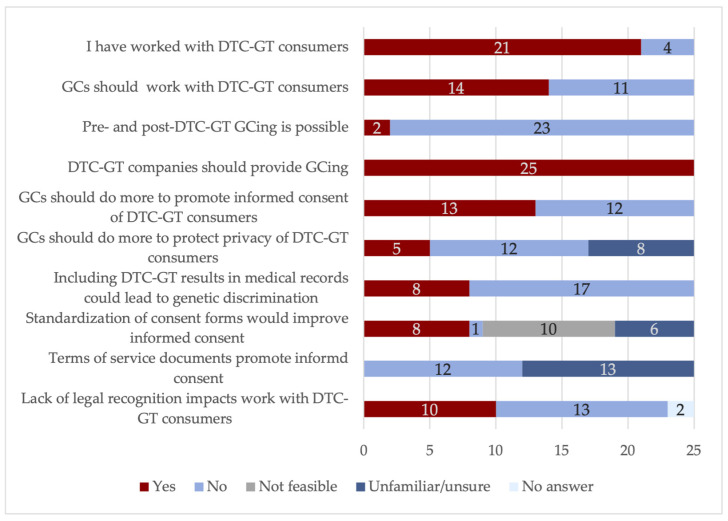
Genetic counselors’ responses to the yes/no survey queries. The survey items covered themes around informed consent, privacy, and the role of GCs with regards to DTC-GTs.

While it is general knowledge that GCs are wary of integrating with DTC-GT results that are not validated, to our knowledge this was the first survey to explicitly interrogate Canadian GCs opinions on DTC-GTs and how their vocation and professional organizations could react to the emerging market. Of the surveyed GCs, the majority (84%, *n =* 21) had experience working with patients who had pursued DTC-GTs (Figure 1), and thus the study results, while non-significant, reflected the views of an experienced population. Indeed, such a number alone represented the growing normalcy of counseling DTC-GT patients within the GCing profession. In a different clinical context, this level of experience working with DTC-GT consumers was a marked change from a 2011 study where only 14% of American GCs had encountered patients who requested the interpretation of DTC-GT results [32], and an increase from a 2016 survey in which 40% of American GCs had provided counseling post-DTC-GT [23]. This growth over time paralleled the expanding DTC-GT industry.

As for concerns regarding consent, the GCs surveyed here did not believe the tests promoted the informed consent of patients; terms and service documents poorly described the tests’ benefits, limitations, and methodology to patients, and therefore failed to adequately disclose the risks to consumers (Table 1). However, there was consensus in the literature that a healthcare professional should be involved with DTC-GT consumers, despite GCs’ opinions on their professional involvement. Among academics [3,8,9,18,22,23,24] and academic institutions [21,25,26,27,33,34], there have been many calls for healthcare professionals to be involved with DTC-GT results. Surveyed public health professionals in Europe have similarly expressed that health professionals should be involved in the process of taking a DTC-GT [35].

Overall, GCs recognize the problems with DTC-GT consent documents (in accordance with the previous findings [32]) and the harm to informed consent that may arise from the lack of healthcare professionals mediating access to tests. However, they do not appear to agree on their professional duties in response and are wary of misusing clinical time and resources (Table 1, Figure 1). However, there is a clear perception among the GC community for consumers to receive adequate information to give informed consent. GCs already provide post-DTC-GT counseling to consumers on an ad hoc basis (i.e., when consumers are referred to their services or contact GCing clinics directly), but there exists no professional imperative to offer counseling to DTC-GT consumers, nor to consumers of any other novel DTC-GT genetic technologies.

The results of this preliminary study have demonstrated that most Canadian GCs are highly concerned about the privacy implications of DTC-GTs (Table 2). GCs perceive privacy issues mainly around the sale of aggregate data and the retention of samples, as well as the anonymization of samples. While many GCs expressed interest in implementing independent security auditing, company registration with a government agency, and data encryption, in contrast to their views on promoting informed consent, most GCs did not believe it is within their professional purview to do more to protect the privacy of DTC-GT consumers (Table 2, Figure 1). GCs reported that such actions would not be feasible, with a minority listing that it is beyond the scope of GCing and that such interventions would be a misuse of clinical time (Table 2). Many professional organizations have written about the necessity for privacy of genetic information, including the European Society of Human Genetics [36], the American Society of Human Genetics [37], the Canadian Medical Association [27], and the American Medical Association, which has called for privacy regulations for DTC-GTs [38].

In terms of actionable change, however, GCs are content to take indirect action to improve consumer informed consent and privacy (Table 2). They reported that they are equipped with the necessary training and education to provide informed consent to people who pursue DTC-GTs, which is a marked change from previous surveys, which found that GCs did not feel comfortable providing counseling to DTC-GT consumers due to a lack of knowledge (59.7% of *n* = 482, self-reported [23]).

This survey confirmed the finding from Hsieh et al. (2021) that GCs hold negative views towards DTC-GTs [23]. Only 56% of the surveyed GCs believed patients who pursue DTC-GTs are within the professional scope of GCs (Figure 1), and GCs are divided as to whether DTC-GT consumers should constitute patients under their care (Table 3). Indeed, all the surveyed GCs were in agreement that DTC-GT companies should provide in-house GCing, since the DTC-GT industry has sufficient funds to hire in-house counselors, and access issues and a lack of GCs would create barriers to providing counseling (Table 3). GCs also indicated that using clinical GCs to counsel DTC-GT consumers could help DTC-GT companies avoid taking accountability for their services (Table 3). This unanimous agreement that DTC-GT companies should offer in-house counseling mirrors the previous findings from a survey conducted through the NSGC [23].

At a broad level, GCs are amenable to producing more education materials for promoting public education on DTC-GTs and are also interested in advocating for reform through the CAGC (Table 3). GCs are well versed in communication and are highly trained in the psychosocial aspects of counseling, making them well positioned to produce such materials for the broader public’s use. Collaboration with governmental bodies or consultation with DTC-GT companies directly could be an elegant solution to some of the major privacy concerns GCs perceive in DTC-GTs. With their deep understanding of the technical and psychosocial consequences of privacy breaches in handling sensitive genetic data, GCs are uniquely trained to be of service in this area. A systematic review of the European general publics’ view of DTC-GTs identified educational materials and programs as key tools to support the capacity of individuals to make informed health decisions based on genetic information [39], and so enlisting GCs to produce such materials could similarly benefit Canadians.

It is worth noting that, of those who had worked with DTC-GT consumers, GCs did not view their lack of professional legal recognition as an impediment, suggesting that GCs do not feel constrained by the lack of delegated acts in this area of their work (Figure 1). Only 13/25 GCs anticipated increased collaboration with healthcare professionals regarding the impact of DTC-GTs (Table 3), which may imply that GCs do not anticipate undertaking follow-up genetic testing from DTC-GT with other healthcare colleagues.

As an interesting final note from the survey, the GCs surveyed here did not anticipate that the inclusion of DTC-GT results in medical records would lead to genetic discrimination (Figure 1). This finding was in apparent opposition to the literature, where scholars have noted that the discrimination inherent in DTC-GTs through insensitive algorithms leads to inaccurate or misleading information, which should not be consulted by healthcare professionals for diagnostic purposes [40]. While not directly leading to genetic discrimination, since DTC-GTs carry questions of scientific validity [11,12,41], consulting such data in healthcare decisions or even treatment could lead to substandard care in racialized populations, and thus, indirectly, genetic discrimination. This has grave potential for harming individuals and racialized populations, when health decisions are made from misleading or inaccurate genetic data [40]. GCs offering DTC-GT counseling to racialized peoples would be a powerful impediment against such alarming consequences.

Despite the professional hesitation to engage with patients who have pursued DTC-GTs, GCs hold the most potential for aiding this novel patient group. DTC-GT consumers have the potential to overwhelm healthcare providers, and physicians have reported a lack of confidence in counseling DTC-GT patients [42] with their limited training in genetics [43]. Indeed, recent studies have reported a 26% error rate in physician interpretation of DTC-GT genetic results [44]. An error rate of over 25% remains concerning and would hold serious consequences for patients if diagnoses are delayed and necessary confirmatory tests are withheld. In contrast, GCs are uniquely trained in psychosocial counseling as well as genetics to hear and address the emotional values and technical questions that may emerge during the DTC-GT odyssey, and to refer patients to geneticists or other physicians for clinical validation if necessary. In light of these factors, GCs are the leading healthcare provider with the knowledge and psychosocial skills to counsel DTC-GT consumers.

### 3.2. Survey Demographics

**Figure 2:**
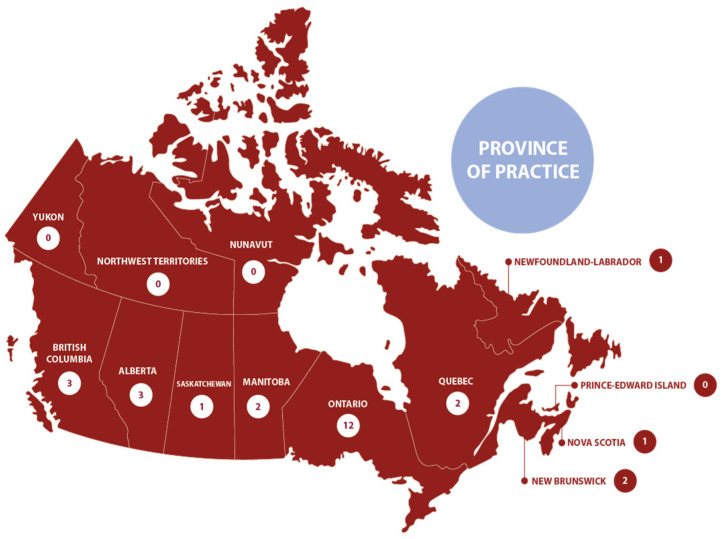
Representation of the GC participants across Canada. Most of the participants practice in Ontario, although there was representation from most provinces in the country.

GCs from 9 out of the 13 Canadian provinces and territories responded to this survey, with the majority practicing in Ontario (Figure 2). A total of 22 GCs reported their current positions as clinical, with representation from GCs working in laboratory settings, research, academic or education, and industry. The participants of this survey all graduated after 1992, with most of the participating GCs having graduated after 2010.

### 3.3. Practice Implications

In light of these survey findings, ideas around GCing and DTC-GT consumers must be revisited. Notably, the CAGC and NSGC recommend that DTC-GT consumers seek pre- and post-DTC-GT counseling [21,26]. However, there is dissonance between the CAGC recommendation and the results of this survey, wherein GCs reported they have adequate training to counsel DTC-GT consumers but do not wish to engage directly with the additional cohort of patients.

To preface, DTC-GT consumers present novel challenges to Canadian GCs who chose to offer them counseling. DTC-GT consumers risk receiving distressing results that could identify unexpected family relations or ancestry information, or even anxiety-inducing health information with unclear clinical utility or unconfirmed gene-to-disease-phenotype correlation [45]. Indeed, non-profit organizations have sprung up to support consumers who have received distressing ancestry test results [46], and third party apps and websites are emerging to help consumers ‘demystify’ DNA results [47]. Ergo, GCs amenable to counseling DTC-GT consumers will have to adapt their services to meet the needs of this distinctive patient group as they strive to meet their professional obligations.

On a fundamental level, GCs may feel they are unable to satisfy a basic requirement of counseling when engaging with DTC-GT consumers: that of the duty to inform. Defined as providing non-directive information and risks on the diagnosis, treatment, consequences, risks, alternatives, and patient support groups, GCs may struggle to meet this obligation when counseling DTC-GT consumers due to their lack of control over and/or knowledge of the tests. The process of providing informed consent would be hindered, as DTC-GT companies rarely provide detailed information on the methodology and counselors would have to contend with a lack of information on the tests’ clinical validity and utility, and GCs would face similar barriers when discussing privacy policies and data storage, use, sharing, and retention with consumers. As for post-DTC-GT counseling, DTC-GT companies may have varying incidental findings policies, potentially returning findings to anxious customers ill prepared to deal with the information. With the varying methodologies and coverage of DTC-GTs [48], GCs may struggle to counsel anxious patients on results that may be false positives, or provide warning of false negatives in this context. Since false positives and negatives are common [19] and can severely impact an individual’s medical choices and health behaviours [12,18], without further medical verification of suspicious test results, GCs may feel their duty to inform patients has not been met. Additionally, GCs who provide counseling to patients who pursue DTC-GTs may benefit from additional support in how to address sensitive topics, such as the racial bias in tests when counseling racialized peoples with confusing or vague results. As the field of genomics faces epistemic and construct validity challenges owing to a lack of diversity in studied populations [49], GCs need to be aware of the consequences this may hold for genetic testing and DTC-GTs. GCs will, thus, have to use their professional judgement to decide if follow-up clinical validation is required, which will be impacted by the availability of resources in the given health jurisdiction and will potentially incur additional costs for the health system [10,12,15,16].

As an additional consideration, GCs broadly must be prepared to adapt to DTC-GT patients’ specific values. Unlike patients in the medical system seeking counseling to inform future genetic tests or to discuss the results of medically accredited tests, DTC-GT patients may approach the counseling session with unfounded beliefs about what information the tests can provide. GCs must be prepared to discuss the limits of DTC-GTs to potentially frustrated and confused patients, as the debate over whether DTC-GTs empower patients’ autonomy to make informed health choices or change health behaviours may affect how patients who have pursued DTC-GTs approach the counseling session.

On a practical level, GCs may need to reassess the provision of in-person care, given the rising number of DTC-GT patients. To address the accessibility concerns of GCing, this survey interrogated GC’s views on virtual conferences. Here, a minority of GCs reported interest in providing intra- and inter-provincial virtual conferences (which would have no legal consequences given that GCing is not a licensed profession), while an equal number listed any such action as not feasible (Table 3). Within the literature, there is debate around implementing tele- or electronic-based counseling, which could open access to genetics services to rural populations, help triage available GCing resources, and decrease costs while, in theory, reducing wait times for medical decision making [50]. In contrast, it has also been argued that offering remote GCing services carries potential to increase patient stress and anxiety over results, increase potential distractions and misunderstanding, and lead to inefficient counseling, as GCs may miss patient nonverbal cues and face additional challenges in providing emotional support [22,50]. However, to meet the needs of a rising DTC-GT patient population, virtual counseling options may be critical for ensuring access to GCing for the appropriate patients to receive care.

When directly asked at what stage in the DTC-GT odyssey they would prefer to offer counseling, GCs reported a preference to provide counseling after receiving the DTC-GT results, presumably to help with results interpretation (ranked as the leading concern of patients who had sought DTC-GTs) (Table 3). However, we contend that preemptive action to promote informed consent could help reduce the number of DTC-GT consumers seeking results interpretation by promoting consumers’ knowledge of the testing risks and benefits before they take a DTC-GT. This work, stemming from the theory of harm reduction, could intercept downstream consumer anxieties before they manifest through anticipatory education, and would, thus, represent an efficient use of GCs’ limited time and resources.

To satisfy this goal, in lieu of individual-oriented consent processes, perhaps alternative media forms, such as short videos or more graphic representations of the ethical and scientific concepts embedded in DTC-GTs, would attract consumers’ attention and promote informed consent. After reading/viewing these materials, consumers could be asked to take a quick quiz to demonstrate their understanding as evidence of informed consent before finalizing the purchase of a DTC-GT. These educational materials could also be adapted for the type of DTC-GT being purchased. The extent of material for consumer review and the length of the quiz could be stratified according to the severity of the medical risks interrogated by the test, similar to how the FDA ranks DTC-GTs for review based on the medical purpose risk and the likelihood of the results impacting medical care [51].

This could be an area that the CAGC or individual GCs could consult to help design educational materials for both the public and other healthcare practitioners that adequately convey technical concepts and prepare consumers for the implications of the test results. The results of this survey suggested that GCs would be open to producing educational materials on DTC-GTs and collaboration projects with the DTC-GT industry to promote informed consent, and the funds resulting from such collaborations could be further used for GC research or undertaking initiatives relating to GC professional recognition (Table 3). While there is no established industry standard for informed consent, Canadian GCs have the knowledge, training, and expertise in communication, counseling, and genetics to guide the development of informed consent materials for DTC-GT companies as a primary mechanism to improve the pre-DTC-GT consent process. When asked to rank what they think their overall engagement with the DTC-GT industry ought to be, 76% of surveyed GCs indicated interest in being involved, and so these measures could very well be a feasible first step (Table 3).

While the GCs surveyed here did not support a DTC-GT counseling role for their profession, in the literature, Wade and Wilfond (2006) suggested that GCs do have a duty to interpret DTC-GT results. These authors suggested that the duty of care GCs owe to their patients should extend to DTC-GT consumers, which includes referrals to other physicians or specialists [52]. In their view, any DTC-GT consumer who requests counseling after taking a test should be seen by a GC, or a physician in the case where the GC does not feel confident providing results interpretation [52]. In the United States, using the NSGC Code of Ethics, others have similarly argued that GCs have a professional duty to offer pre- and post-genetic test counseling under the premise that the NSGC strives to be the healthcare resource that members of the public access for questions about genetic testing [53]. Ideally, this would be the case in the Canadian healthcare context, and the CAGC does call for pre- and post-DTC-GT genetic counseling [26]. However, after over three years of battling COVID-19, and with the low number of practicing GCs, Canada’s healthcare infrastructure is not well equipped to deal with the high influx of DTC-GT patients should such a counseling duty be codified. GCs themselves do not see this as their responsibility or their obligation, as demonstrated by the survey results.

DTC-GTs and other emerging biotechnologies will continue to enact changes in the role of GCs and other healthcare practitioners as the marketspace of these tests continues to grow. Here, GCs reported increased collaboration with primary care physicians and other healthcare practitioners, increased work beyond their traditional clinical boundaries, and increased advocacy work as a direct consequence of DTC-GTs (Table 3). With their unique combination of psychosocial counseling knowledge and genetic expertise, this population of Canada’s healthcare workers are ready and able to meet the challenges of DTC-GTs, and lessons learned will be of use for other factions of the healthcare sector as biotechnologies continue to emerge.

### 3.4. Research Recommendations

This preliminary survey described the sentiments of Canadian GCs towards counseling patients who pursue DTC-GTs and the many implications this additional role holds for the vocation. While this survey gathered rich data, the participant response rate was not high enough to reach a statistical significance, and thus the results may not be generalizable across the entire GC vocation. To continue to clarify GC opinions on DTC-GTs and their professional obligations, interviews with GCs in clinical, academic, and industry roles could shed valuable insight on the feasibility of the proposed changes to the novel GC roles proposed here. The role of the GC is facing new challenges with the expansion of biotechnologies into the hands of consumers and additional research in this area will help to identify the most efficient use of the limited time and resources of Canadian GCs.

## 4. Conclusions

Overall, the results from this pilot survey suggest that Canadian GCs remain fundamentally divided over several key issues regarding consumer informed consent and privacy, and that GCs additionally remain divided over to the appropriate role individual GCs and the GCing profession ought to play in this age of unprecedented public access to novel biotechnologies.

DTC-GTs could pose a potential new route for a preventative model of healthcare, with citizens actively participating in health monitoring through these commercialized personalized medicine products. This model could save the public health system innumerable costs if the tests are scientifically accurate, clinically valid, and function in diverse populations. DTC-GTs could pose a powerful knowledge translation tool for increasing science literacy and empowering patients in the genomics era, but without increased intervention by healthcare professionals, the tests remain unregulated with an untapped potential.

## Figures and Tables

**Table 1:** Responses to the survey items regarding the informed consent process of DTC-GTs.

Question	Response	
Do DTC-GT terms of service documents sufficiently describe the limitations of DTC-GTs?	20% (*n* = 5): Too varied	
	0% (*n =* 0): Clearly describe	
	32% (*n* = 8): Poorly describe	
	0% (*n =* 0): Do not describe	
	48% (*n* = 12): Unfamiliar	
Does the language in DTC-GT terms of service documents sufficiently promote informed consent?	0% (*n* = 0): Yes	
	48% (*n* = 12) No	
	52% (*n* = 13): Unfamiliar	
How would you rate the following statement: DTC-GTs remove GCs from discussions of genetic health data, which negatively impacts consumer informed consent.	8% (*n* = 2): Strongly agree	
	52% (*n* = 13): Agree	
	24% (*n* = 6): Disagree	
	16% (*n* = 4) Disagree	
	0% (*n* = 0): Strongly disagree	
Do you interpret the Canadian Association of Genetic Counsellors (CAGC) Code of Ethics tenant to “promote awareness of the roles of medical genetics professionals” to include collaboration with the DTC-GT industry?	76% (*n* = 19): Yes	
	24% (*n* = 6): No	
	Why no?	8% (*n* = 2): Beyond the scope of practice
		12% (*n* = 3): Conflict of interest
	Why yes?	4% (*n* = 1): No response
		64% (*n* = 16): Would allow for evaluation of DTC-GTs
		56% (*n* = 14): Would allow for standardized informed consent
		20% (*n* = 5): DTC-GT company funding could facilitate research and development in GCing
The CAGC Code of Ethics states that GCs should: “... promote awareness of the roles of medical genetics professionals through activities such as participation in multi-disciplinary teams, providing public education, contributing to policy-making and provincial/national consultation”. Do you think that “providing public education” should include information on DTC-GTs?	84% (*n* = 21): Yes	
	16% (*n* = 4): No	
Do you believe the vocation of GCing needs to do more to protect the informed consent of DTC-GT customers compared to traditional patients seeking counseling?	52% (*n* = 13): Yes	
	48% (*n* = 12): No	
	Why no?	20% *(n* = 5): DTC-GT is not medically necessary; does not merit intervention from healthcare professionals
		36% (*n* = 9): Inefficient use of clinical time and resources
	Why yes?	0% (*n* = 0): GCs are not adequately trained to counsel DTC-GT customers on informed consent
		44% (*n* = 11): The tests contain significant limitations which may be unclear to consumers
		44% (*n* = 11): The absence of healthcare professionals creates additional challenges to informed consent that merit attention
		32% (*n* = 8): There is limited information about the accuracy of DTC-GT results
DTC-GTs continue to improve the quality and accuracy of results every year. How does the continual improvement of DTC-GT results impact GCing?	24% (*n* = 6): Multi-disciplinary medical collaborations	
	40% (*n* = 10): Longer relationship with DTC-GT customers with changing results	
	24% (*n* = 6): Diverting clinical resources with expanding knowledge demands	
	56% (*n* = 14): Does not impact GCing; field evolving	

**Table 2:** Responses to the survey items regarding the privacy of DTC-GTs.

Question		Response
How concerned are you with the privacy policies of DTC-GT companies in Canada? Scale of 1 (unconcerned)–5 (concerned)		4% (*n* = 1): 1 (unconcerned)
		8% (*n* = 2): 2
		28% (*n* = 7): 3
		52% (*n* = 13): 4
		8% *(n =* 2): 5 (concerned)
		Average response: 3.52 (concerned, standard deviation = 0.92)
Where do you perceive privacy issues in DTC-GTs?		84% (*n =* 21): Sale of aggregated data
		72% (*n =* 18): Retention of samples
		40% (*n =* 10): Anonymization of genetic data
		4% (*n* = 1): Canadian Federal Law sufficiently protects consumer privacy
		12% (*n =* 3): DTC-GTs adequately safeguard personal genetic security
Do you believe the vocation of GCing needs to do more to protect the privacy of DTC-GT customers compared to traditional patients seeking counseling?		48% (*n =* 12): No
		20% (*n =* 5): Yes
		32% (*n =* 8): Unsure
	Why no?	32% (*n =* 8): Intervention not feasible
		24% (*n =* 6): Beyond the scope of GCing
		24% (*n =* 6): Intervention was a misuse of clinical time
		20% (*n =* 5): The lack of regulation of DTC-GT company privacy policies is concerning
	Why yes?	4% (*n =* 1): Since informed consent is difficult to achieve without the mediation of a healthcare professional, privacy policies must be closely monitored
		20% (*n* = 5): The lack of regulation of DTC-GT company privacy policies is concerning

**Table 3:** Responses to the survey items regarding the broader potential policies GCs could implement in response to the rise of DTC-GTs.

Question		Response
Generally speaking, how heavily do you think the GCing vocation should be involved with DTC-GTs? 1–not at all involved, 5–heavily involved.		0% (*n* = 0): 1
		24% (*n* = 6): 2
		36% (*n* = 9): 3
		32% (*n* = 8): 4
		8% (*n* = 2): 5
How would you rate the following statement: DTC-GTs are sufficiently regulated.		0% (*n* = 0): Strongly agree
		0% (*n* = 0): Agree
		24% (*n* = 6): Neutral
		76% (*n* = 19): Disagree
		0% (*n* = 0): Strongly disagree
What level of involvement should the CAGC hold with respect to DTC-GT evaluation?		20% (*n* = 5): Grade tests (maintain a list on the website)
		12% (*n* = 3): Recommend tests (through the website)
		16% (*n* = 4): Accredit tests (label products)
		48% (*n* = 12): Currently not feasible for the CAGC to be involved in this process
		56% (*n* = 14): The CAGC should not attempt to evaluate DTC-GTs
Do you believe it is feasible to offer pre- and post-clinical genetic counseling to DTC-GT customers?		8% (*n* = 3): Yes
		92% (*n* = 23): No
	Why no?	72% (*n* = 18): Not enough GCs
		72% (*n* = 18): Should be the responsibility of DTC-GT companies
Does pre- or post-DTC-GT counseling have the potential for the most impact on consumers?		28% (*n* = 7): Before
		44% (*n* = 11): After
		28% (*n* = 7): Not feasible to offer any counseling
Which of the following methods could alleviate the burden of DTC-GT counseling?		40% (*n* = 10): Virtual conferences within province
		40% (*n* = 10): Virtual conferencing within the country
		56% (*n* = 14): Online resources managed by GCs
		40% (*n* = 10): Not feasible to offer any counseling
Given the high demand for counseling for DTC-GT customers, what is currently feasible for the CAGC to offer?		48% (*n* = 12): Establish a special interest group (SIG) at the annual CAGC meeting to develop policy review of DTC-GTs
		44% (*n* = 11): Organize seminars for primary care physicians or other healthcare professionals to prepare them to offer counseling of DTC-GT results
		60% (*n* = 15): Advocate for federal regulation of DTC-GTs
		68% (*n* = 17): Produce informative materials
How do you see the current role of genetic counselors changing with regards to the DTC-GT industry?		60% (*n* = 15): Increased collaboration with primary care physicians
		52% (*n* = 13): Increased collaboration with other healthcare professionals
		52% (*n* = 13): Increased work outside clinical roles
		52% (*n* = 13): Increased advocacy work
		24% (*n* = 6): GCs should focus on clinical patients rather than DTC-GT customers
How would you respond to the following statement: It is the responsibility of DTC-GT companies to provide counseling for consumers rather than clinical GCs		100% (*n* = 25): Yes
		0% (*n* = 0): No
	Why yes?	56% (*n* = 14): Too few accredited genetic counselors across Canada to provide this service
		56% (*n* = 14): Uneven distribution of clinical GCs across Canada creates access issues
		60% (*n* = 15): Since tests are not medically necessary, customers do not merit access to a limited pool of GCs
		40% (*n* = 10): DTC-GT customers are not patients of the healthcare system
		84% (*n* = 21): The DTC-GT industry has sufficient resources to recruit GCs
		40% (*n* = 10): Offsetting the DTC-GT counseling responsibility helps the industry avoid accountability

## Data Availability

The data presented in this study are available upon request from the corresponding author. The data are not publicly available due to confidentiality concerns for the survey participants.
